# Association analysis of chromosome 1 migraine candidate genes

**DOI:** 10.1186/1471-2350-8-57

**Published:** 2007-08-29

**Authors:** Francesca Fernandez, Robert P Curtain, Natalie J Colson, Micky Ovcaric, John MacMillan, Lyn R Griffiths

**Affiliations:** 1 Genomics Research Centre, School of Health Science, Griffith University, Gold Coast, Queensland, Australia; 2 Queensland Clinical Genetics Service, Royal Children's Hospital Health Service District, Brisbane, Queensland, Australia

## Abstract

**Background:**

Migraine with aura (MA) is a subtype of typical migraine. Migraine with aura (MA) also encompasses a rare severe subtype Familial Hemiplegic Migraine (FHM) with several known genetic loci. The type 2 FHM (FHM-2) susceptibility locus maps to chromosome 1q23 and mutations in the ATP1A2 gene at this site have recently been implicated. We have previously provided evidence of linkage of typical migraine (predominantly MA) to microsatellite markers on chromosome 1, in the 1q31 and 1q23 regions. In this study, we have undertaken a large genomic investigation involving candidate genes that lie within the chromosome 1q23 and 1q31 regions using an association analysis approach.

**Methods:**

We have genotyped a large population of case-controls (243 unrelated Caucasian migraineurs versus 243 controls) examining a set of 5 single nucleotide polymorphisms (SNPs) and the Fas Ligand dinucleotide repeat marker, located within the chromosome 1q23 and 1q31 regions.

**Results:**

Several genes have been studied including membrane protein (ATP 1 subtype A4 and FasL), cytoplasmic glycoprotein (CASQ 1) genes and potassium (KCN J9 and KCN J10) and calcium (CACNA1E) channel genes in 243 migraineurs (including 85% MA and 15% of migraine without aura (MO)) and 243 matched controls. After correction for multiple testing, chi-square results showed non-significant P values (P > 0.008) across all SNPs (and a CA repeat) tested in these different genes, however results with the KCN J10 marker gave interesting results (P = 0.02) that may be worth exploring further in other populations.

**Conclusion:**

These results do not show a significant role for the tested candidate gene variants and also do not support the hypothesis that a common chromosome 1 defective gene influences both FHM and the more common forms of migraine.

## Background

Migraine with aura (MA) is defined as the more severe subtype of typical migraine and is associated with focal neurological symptoms such as altered vision accompanying the usual symptoms of migraine [1]. Migraine with aura (MA) also contains a rare severe subtype of Familial Hemiplegic Migraine (FHM), which is accompanied by migraine headache and quite severe associated neurological symptoms [1]. The type 2 FHM (FHM-2) susceptibility locus maps to chromosome 1q23 and mutations in the ATP1A2 gene at this site have recently been implicated in two FHM pedigrees not showing linkage to the FHM-1 locus on chromosome 19p13 [2]. We have previously provided evidence of linkage to microsatellite markers on chromosome 1 showing evidence of linkage to chromosome 1q31 (C1q31) [3] and also evidence of excess allele sharing in predominantly MA affected pedigrees to the C1q23 genomic region [4]. We have previously also investigated the FHM-2 (ATP1A2) gene located at C1q23 through mutation analysis but found no link between this gene and the common forms of migraine [4].

To further investigate a potential chromosome 1q involvement in migraine, we undertook association analysis of other potential candidate genes that lie within the 1q23 and 1q31 regions on chromosome 1. These include a subunit of the sodium/potassium ATPase, ATP1A4, an integral membrane protein catalytic subunit directly responsible for ion transport such as the active transport of sodium ions in exchange for potassium ions across cell membranes. The electrochemical gradient generated by this exchange is essential for maintaining membrane potentials and for sodium – coupled co-transport of a variety of organic and inorganic molecules [5]. Another gene tested was CASQ1 (Human fast-twitch skeletal Calsequestrin 1), an acid glycoprotein believed to function as a storage protein for calcium in the sarcoplasmic reticulum [6]. This protein binds calcium ions with a high capacity but with a moderate affinity and consequently is able to modulate activity of the ryanodine receptor, controlling muscle calcium homeostasis and thus excitation-contraction coupling [7]. Furthermore, CASQ 1 can regulate the GATA 4-and AP-1-dependent transcriptional events, indicating the existence of sarcoplasmic reticulum nuclear circuits of signal transduction in adult cardiac muscle [8]. Several cardiac disorders notably some congenital heart defects, such as those affecting the patent foramen oval have recently been reported to be comorbid with migraine [9], suggesting a potential common genetic component in these disorders.

Channel mutations or "channelopathies" are increasingly found to be the cause of various neurological disorders in humans [10]. The KCN channels (potassium inwardly-rectifying channel) participate in polarisation of neuronal membrane and the regulation of the action potential, whereby they control neuronal excitability. In this study, the human KCNJ9 and KCNJ10 genes (potassium inwardly-rectifying channel, subfamily J, member 9 and 10) were also investigated. They are members of the G-protein-activated inwardly rectifying potassium (K^+^) (GIRK) channel family responsible for playing a critical inhibitory role in K^+^transport throughout the nervous system, heart, pancreas, brain and kidney [11]. These channels have widespread expression in the central nervous system, helping to regulate extracellular K^+ ^ion concentrations.

We also investigated a CA repeat polymorphism localized 46-kb upstream of the Fas ligand gene (FasL) gene on C1q23. FasL is a transmembrane protein belonging to the tumor necrosis family (TNF, member 6). It is an apoptosis inducing T-cell effector predominantly expressed in activated T lymphocytes, in natural killer cells and in immune privileged sites, such as the testes and eye [12]. FasL is also involved in vascular smooth muscle cell apoptosis, which part of atherosclerotic plaque rupture causing myocardial infarction [13].

Finally, the CACNA1E gene on C1q31 was also tested. This is a second region of interest on C1 for typical migraine, as previous results from our laboratory have also shown linkage to this region [3]. CACNA1E is the alpha 1E subunit of R-type voltage-dependent calcium channels. Notably CACNA1A, the alpha 1A subunit at C19p13 has been implicated in FHM-1 [14]. These subunits form voltage-sensitive calcium channels (VSCC), which mediate the entry of calcium ions into excitable cells. They are also involved in a variety of calcium-dependent processes, including muscle contraction, hormone or neurotransmitter release, gene expression, cell motility, cell division and cell death. Calcium channels containing alpha-1E subunit could be involved in the modulation of firing patterns of neurons which is important for information processing.

Overall this study investigated six genes localized to the chromosome 1q23 and 1q31 regions, five of which contain informative SNPs and one dinucleotide repeat upstream from the FasL gene. The population utilized was a large case-control migraine population – age, sex and ethnically matched.

## Methods

### SNP and dinucleotide polymorphism selection

This study incorporated analysis of a CA dinucleotide repeat polymorphism located 46-kb upstream from the Fas ligand gene (FasL) and also SNP analysis techniques for variants within other candidate genes on chromosome 1q23 and 1q31. SNPs were selected using SNPbrowser™ Software (ABI), Ensembl Genome Browser and NCBI SNP databases. A detailed summary of all variants selected for this study is displayed in Table 1.

**Table 1:** A detailed summary of all variants selected for this study

**GENE**	**Ref SNP ID**	**Chromosome location**	**Coding**	**Alleles**	**Translation**	**AA change**	**Minor allele freq**	**Restriction enzyme**	**Restriction site**	**RE Digest fragement (bp)**
KCNJ10	rs1130183	C1q23.2	yes, exon2	C/T	non-syn	Arg-Cys	T = 0.0240	Ear I	CTCTT**C**	614,376,238,8
KCNJ9	rs2494211	C1q23.2	no,3'-UTR	T/C	Nil	nil	C = 0.28	Rsa I	G**T**AC	382,204,178
ATP1A4	rs6427504	C1q23.3	yes, exon3	T/C	non-syn	Gly-Asp	C = 0.337	Bcc I	CCA**T**C	365,249,116
CASQ1	rs3747623	C1q23.3	no, intron3	T/C	nil	nil	C = 0.481	Hph I	**T**CACC	245,189,116,56
CACNA1E	rs704326	C1q25.3	yes, exon43	G/A	non-syn	Ala-Thr	G = 0.390	AciI	CC**G**C	153,109,44
FasL	nil	C1q25.2	No,3'-UTR	CA rpt	nil					

### PCR conditions

Primer sequences for each SNP and the dinucleotide repeat were designed by utilizing Primer Express™ v 2.0 software (ABI) or obtained from previous publications (see discussion) (Table 2). All PCR reactions for each SNP and the FasL marker utilized final concentrations of MgCl_2,_Primers, dNTPs, 10 × Buffer and *Taq *polymerase with 1.75 mM, 0.2 μM, 200 μM, 1 × and 1U respectively. Approximately 40 ng of DNA per reaction was used in the PCR. Cycling conditions for ATP1A4 and CASQ1 SNPs consisted of 94°C for 4 minutes, then 35 cycles of 94°C for 1 minute, 55°C for 1 minute and 72°C for 30 seconds. The final PCR extension consisted of 72°C for 2 minutes. For KCNJ10 an annealing temperature of 57°C was needed while the FasL marker and CACNA1E SNP utilized a 35 cycle – 60°C annealing step without an extension step incorporated.

**Table 2:** Primer sequences utilized for SNP and CA polymorphism PCR amplication

**Ref SNP ID**	**GENE**	**Recombination mapping position**	**Sequence 5'->3'**
rs1130183	KCNJ10	155.96 – 156.04 cM	F-GCAAGCCCTGCCTCATGA and R-AGGGCATTGGAAGAGAGG
rs2494211	KCNJ9	156.05 – 156.08 cM	F-CAGGCACAGGCAGGAAGAG and R-GTGATTCCAACGCACACTGG
rs6427504	ATP1A4	156.13 – 156.17 cM	F-GCTCCAAATGTCAGGTTCCC and R-GAGTGCTAGGCTGCTGAGCA
rs3747623	CASQ1	156.16 – 156.20 cM	F-CCCCTAGGCTTAATCCAGGG and R-GGGCAGGCAGTGGAGTTTT
Nil	FasL (CA Rpt)	174.93 – 174.95 cM	F-ACCATTTCACTCACACACAAC and R-GGTAAGGGAAGCACAATAACTG
rs704326	CACNA1E	181.83 – 182.42 cM	F-CCTCATTACCTCTGGATTTGACATT and R-CATTCACCAGAGACTGCCGTT

Positioning of genes (cM) based on NCBI Map Viewer using 'deCODE' and 'Genes On Sequence' mapping data

### Genotyping analysis and Restriction Digest conditions

The FasL dinucleotide repeat marker was labelled with FAM fluorescent dye label and following PCR loaded on an ABI310™ Genetic Analyser with Genescan Analysis™ 3.1 software. Genotyping was performed with ABI Genotyper™ 2.1 software.

SNP analysis was performed using restriction enzyme sites overlapping the SNP in question. Digest conditions for all SNPs utilized 10 ul of PCR product and a 10 ul mix of 3U of restriction enzyme with 1 × enzyme buffer and H_2_O. Digest products were loaded and electrophoresed on 3–4% high resolution agarose gels whereby genotyping was performed.

### Statistical data analysis

Genotype and allele frequencies for all SNP variants and the CA repeat were calculated from observed genotype counts. As a statistical control for systematic genotyping error and population stratification, the expected genotype proportions according to the Hardy-Weinberg law were calculated and compared to observed genotypes. Genotype and allele frequencies were initially assessed for association with migraine, then MA and MO subpopulations were assessed, using conventional contingency table analyses incorporating the standard chi-squared test for independence. Genetic risk magnitudes (effect size) were estimated by calculating ORs with 95% confidence intervals.

In total, we investigated the six DNA markers in controls and case groups of the population (MA, MO, Migraine (MO+MA)). Therefore, the standard significance level of 5 % has been adjusted to α = 0.05/6 = 0.008, according the application of Bonferroni correction for multiple testing, to provide evidence for association of the studied polymorphisms with migraine susceptibility.

### Subjects

A cross-sectional association approach was employed, utilizing genomic DNA samples obtained from 243 migraine affected individuals and 243 controls. The populations consisted of Caucasians from general Australian Community. To minimize potential bias from population stratification, the control group was matched for sex, age (+/- 5 years) and ethnicity. Migraine patients were clinically defined and suitably matched non-migraine individuals were obtained for the control population. The subjects were diagnosed for migraine using a detailed questionnaire and clinical neurologist (JM) in accordance with the International Headache Society criteria [1]. The blood samples obtained from patients were collected through the Genomics Research Centre patient clinic and purified DNA from these samples was obtained using standard extraction methods [15].

Individuals were grouped together as total migraine affected, containing both MA and MO diagnosed samples. For further statistical analysis the groups were then split into migraine with aura and migraine without aura populations. The control population consisted of non-migraine affected individuals.

### Ethical approval

This research was reviewed and approved by the Griffith University Human Research Ethics Committee (ethics protocol numberMSC/05/05/HREC) and all subjects participating in the study gave informed consent.

## Results

Six markers localised to the C1q23-C1q31 region covering several genes of interest (ATP1A4, CASQ1, KCNJ9 and J10, FasL and CACNA1E), have been analysed in regard to their potential association with migraine in a large population (243 migraineurs versus 243 healthy individuals). The distribution of all genotypes of the variants in the studied population did not deviate significantly from Hardy-Weinberg Equilibrium (P > 0.05).

Table 3 represents the results of the allelic and genotypic frequency distribution of the studied SNPs. Results showed no significant difference between controls and migraine patients for the allelic frequencies (χ^2 ^= 1.59, 2 df, P = 0.207; χ^2 ^= 0.46, 2 df, P = 0.713; χ^2 ^= 1.06, 2 df, P = 0.496; χ^2 ^= 0.14, 2 df, P = 0.303; χ^2 ^= 1.00, 2 df, P = 0.6 respectively for ATP1A4, CASQ1, KCNJ9, KCNJ10 and CACNA1E). Also there was no significant association for the genotypic frequencies between controls and migraine for any of the studied polymorphisms (P > 0.008), taking into account the Bonferroni corrected significance level (α = 0.008) (Table 3). Stratified analyses of migraine subtypes did not indicate any association specifically attributed to the MA or MO subtype group for both allelic and genotypic frequencies of all the five studied SNPs (P > 0.008), as shown in Table 3. Results from KCNJ10 marker however did show an interesting result for genotypic analysis (χ^2 ^= 7.65, P = 0.02) but there were very low numbers in some of genotype groups, and a larger population would be needed to assess these variations more appropriately (Table 4). When the results were analysed by gender and by subtype of migraine, no significant association was observed for KCNJ10 genotype and allelic distribution (Table 4).

**Table 3:** Distribution of the ATP1A4, CAS Q1, KCNJ9, KCNJ10, and CACNA1E SNPs in migraineurs and controls of original sample

	N	Alleles			Genotypes	
Group	(alleles)	C	T	CC	CT	TT

**ATP 1A4 SNP**	**rs 6427504**					
**Migraine**	**466**	**167 (35.8%)**	**299 (64.2%)**	**30 (12.9%)**	**107 (45.9%)**	**96 (41.2%)**
Male	64	22 (34.4%)	42 (65.6%)	4 (12.5%)	14 (43.8%)	14 (43.8 %)
Female	402	145 (36.1%)	257 (63.9%)	26 (12.9%)	93 (46.3%)	82 (40.8%)
MA	382	139 (36.4%)	243 (63.6%)	24 (12.6%)	91 (47.6%)	76 (39.8%)
MO	74	26 (35.1%)	48 (64.9%)	5 (13.6%)	16 (43.2%)	16 (43.2%)
**Control **	**416**	**167 (40%)**	**251 (60%)**	**38 (18.2%)**	**91 (43.5%)**	**80 (38.3%)**
Male	66	25 (37.9%)	41 (62.1%)	5 (15.2%)	15 (45.5%)	13 (39.3%)
Female	352	142 (40.3%)	210 (59.7%)	33 (18.8%)	76 (43.1 %)	67 (38.1%)
**Total case *vs *control**		***X***^2^*** = 1.59 ***	***P = 0.207***		***X***^2^*** = 2.39 ***	***P = 0.302***
*Subtype MA vs cont:*		***X***^2^*** = 1.07 ***	***P = 0.30***		***X***^2^*** = 2.46 ***	***P = 0.29***
*Subtype MO vs cont*		***X***^2^***= 0.61***	***P = 0.43***		***X***^2 ^***= 0.59***	***P = 0.74***

	N	Alleles			Genotypes	

Group	(alleles)	C	T	CC	CT	TT

**CAS Q1 SNP **	**rs 3747623**					
**Migraine**	**446**	**288 (63.2%)**	**168 (36.8%)**	**93 (40.8%)**	**102 (44.7%)**	**33 (14.5%)**
Male	68	45 (66.2%)	23 (33.8%)	16 (47.1%)	13 (38.2%)	5 (14.7 %)
Female	388	243 (62.6%)	145 (37.4%)	77 (39.7%)	89 (45.9%)	28 (14.4%)
MA	376	235 (62.5%)	141 (37.5%)	76 (40.4%)	83 (44.1%)	29 (15.4%)
MO	66	42 (63.6%)	24 (36.4%)	12 (36.4%)	18 (54.3%)	3 (9.1%)
**Control **	**430**	**281 (65.3%)**	**149 (34.7%)**	**89 (41.4%)**	**103 (47.9%)**	**23 (10.7%)**
Male	66	38 (59.4%)	26 (40.6%)	11 (34.4%)	16 (50%)	5 (15.6%)
Female	352	243 (66.4%)	123 (33.6%)	78(47.1%)	87 (38.2 %)	18 (14.7%)
**Total case *vs *control**		***X***^2^*** = 0.46***	***P = 0.496***		***X***^2^*** = 1.48 ***	***P = 0.473***
*Subtype MA vs cont:*		***X***^2^*** = 0.71 ***	***P = 0.40***		***X***^2^*** = 2.07 ***	***P = 0.36***
*Subtype MO vs cont*		***X***^2^*** = 0.07 ***	***P = 0.78***		***X***^2^*** = 0.51 ***	***P = 0.77***

	N	Alleles			Genotypes	

Group	(alleles)	C	T	CC	CT	TT

**KCNJ9 SNP **	**rs 2494211**					
**Migraine**	**466 **	**322 (66.5%)**	**162 (33.5%)**	**113 (46.7%)**	**96 (39.7%)**	**33 (13.6%)**
Male	70	41 (58.6%)	29 (41.4%)	12 (34.3%)	17 (48.6%)	6 (17.1 %)
Female	414	281 (67.9%)	133 (32.1%)	101 (48.8%)	79 (38.2%)	27 (13%)
MA	392	262 (66.8%)	130 (33.2%)	94 (48%)	74 (37.8%)	28 (14.2%)
MO	74	48 (64.9%)	26 (35.1%)	15 (40.5%)	18 (48.6%)	4 (10.9%)
**Control **	**486**	**308 (63.4%)**	**178 (36.6%)**	**95 (39.1%)**	**118 (48.6%)**	**30 (12.3%)**
Male	70	41 (58.6%)	29 (41.4%)	11 (31.4%)	19 (54.3%)	5 (14.3%)
Female	416	267 (64.2%)	149 (35.8%)	84 (40.4%)	99 (47.6 %)	25 (12%)
**Total case *vs *control**		***X***^2^*** = 1.06 ***	***P = 0.303***		***X***^2^*** = 3.96***	***P = 0.138***
*Subtype MA vs cont:*		***X***^2^*** = 1.14 ***	***P = 0.28***		***X***^2^*** = 5.19***	***P = 0.07***
*Subtype MO vs cont*		***X***^2^*** = 0.06***	***P = 0.80***		***X***^2^*** = 0.08 ***	***P = 0.96***

	N	Alleles			Genotypes	

Group	(alleles)	C	T	CC	CT	TT

**KCNJ10 SNP**	**rs1130183**					
**Migraine**	**440**	**408 (92.7%)**	**32 (7.3%)**	**189 (85.9%)**	**30 (13.6%)**	**1 (0.5%)**
Male	66	59 (89.4%)	7 (10.6%)	26 (78.8%)	7 (21.2%)	0
Female	374	349 (93.3%)	25 (6.7%)	163 (87.2%)	23 (12.3%)	1 (0.5%)
MA	356	332 (93.3%)	24 (6.7%)	155 (87.1%)	22 (12.4%)	1 (0.6%)
MO	66	63 (95.5%)	3 (4.5%)	30 (90.9%)	3 (9.1%)	0
**Control **	**422 **	**394 (93.4%)**	**28 (6.6%) **	**189 (89.6%)**	**16 (7.6%)**	**6 (2.8%)**
Male	62	57 (91.9%)	5 (8.1%)	27 (87.1%)	3 (9.7%)	1 (3.2%)
Female	360	337 (93.6%)	23 (6.4%)	162 (90%)	13 (7.2 %)	5 (2.8%)
**Total case *vs *control**		***X***^2^*** = 0.14***	***P = 0.713***		***X***^2^*** = 7.65***	***P = 0.02***
*Subtype MA vs cont:*		***X***^2^*** = 0 ***	***P = 0.95***		***X***^2^*** = 5.12 ***	***P = 0.08***
*Subtype MO vs cont*		***X***^2^*** = 0.42 ***	***P = 0.52***		***X***^2^*** = 1.02 ***	***P = 0.59***

	N	Alleles			Genotypes	

Group	(alleles)	A	G	AA	AG	GG

**CACNA1E SNP**	**rs 704326**					
**Migraine**	464	**384 (83%)**	**80 (17%)**	**154 (66%)**	**76 (33%)**	**2 (1%) **
Male	142	134 (94.4%)	8 (5.6%)	63 (88.7%)	8 (11.3%)	0
Female	308	240 (77.9%)	68 (22.1%)	89 (57.8%)	62 (40.3%)	3 (1.9%)
MA	276	224 (81%)	52 (19%)	88 (64%)	48 (35%)	2 (1%)
MO	188	160 (85%)	28 (15%)	66 (70%)	28 (30%)	0
**Control **	424	**341 (80%)**	**83 (20%) **	**132 (63%)**	**77 (36%)**	**3 (1%) **
Male	116	101 (87.1%)	15 (12.9%)	43 (74.1%)	15 (25.9%)	0
Female	308	240 (77.9%)	68 (22.1%)	89(57.8%)	62 (40.3 %)	3 (1.9%)
**Total case *vs *control**		***X***^2^*** = 1.00 ***	***P = 0.6***		***X***^2^*** = 0.81***	***P = 0.37***
*Subtype MA vs cont:*		***X***^2^*** = 0.06 ***	***P = 0.80***		***X***^2^*** = 0.09***	***P = 0.96***
*Subtype MO vs cont*		***X***^2^*** = 1.92 ***	***P = 0.16***		***X***^2^*** = 2.78 ***	***P = 0.25***

MO, migraine without aura; MA, migraine with aura

**Table 4:** Chi-squared (χ ^2^) analysis of all migraine groups against controls for the KCNJ10 SNP (rs1130183) polymorphism

	**Genotypic frequencies**	**Allelic frequencies**
Migraine *vs *Control	***X***^2^*** = 7.65 P = 0.02***	***X***^2^*** = 0.14 P = 0.71***
MA *vs *Control	***X***^2^*** = 5.12 P = 0.08***	***X***^2^*** = 0 P = 0.95***
MO *vs *Control	***X***^2^*** = 1.02 P = 0.59***	***X***^2 ^*** = 0.42 P = 0.52 ***
Female Migraine *vs *Female Control	***X***^2^*** = 5.32 P = 0.07***	***X***^2^*** = 0.03 P = 0.87***
Male Migraine *vs *Male Control	***X***^2^*** = 2.56 P = 0.28***	***X***^2^*** = 0.24 P = 0.62***

As shown in Table 5, the frequencies of the two major alleles of the FasL CA repeat (alleles, 3 and 4), were found to make up nearly 99 % of the alleles in both the control and migraineur groups. This finding is similar to that reported by Zayas et al. (2001) in 139 unrelated healthy individuals. Results for the marker showed no significant difference for the FasL polymorphism in controls *versus *MA, MO or combined (MA+ MO) groups (χ^2 ^= 5.93, 4 df, P = 0.204; χ^2 ^= 7.55, 4 df, P = 0.109; χ^2 ^= 0.94, 4 df, P = 0.919, respectively).

**Table 5:** Distribution of the FasL (CA repeats) in migraineurs and controls of original sample

			**Alleles **			
Alleles	N	11	12	13	14	15

**Migraine**	**464**	**1 (0.2%)**	**2 (0.4%) **	**228 (49.1%) **	**231 (49.8%) **	**2 (0.4%)**
Male	64	1 (1.6%)	0	27 (42.2%)	35 (54.7%)	1 (1.6 %)
Female	400	0	2 (0.5%)	201(50.3%)	196 (49%)	1 (0.3%)
MA	376	1 (0.3%)	2 (0.5%)	190 (50.5%)	182 (48.4%	1 (0.3%)
MO	88	0	0	38 (43.2 %)	49 (55.7%)	1 (1.1%)
**Control **	**552**	**1 (0.2%)**	**0**	**262 (47.5%)**	**280 (50.7%)**	**9 (1.6%) **
Male	88	0	0	213 (46.3%)	241 (52.4%)	5 (15.6%)
Female	460	1 (0.2%)	0	47 (53.4%)	39 (44.3 %)	2 (2.3%)
**Total case *vs *control**			***X***^2^*** = 5.93 ***	***P = 0.204***		
*Subtype MA vs cont:*			***X***^2^*** = 7.55 ***	***P = 0.109***		
*Subtype MO vs cont*			***X***^2^*** = 0.94 ***	***P = 0.919***		

MO migraine without aura, MA migraine with aura

## Discussion

The C1q23 region covers a large genetic distance of 1–3 cytogenetic bands, is greater than 12 MB in length and contains a high density of genes in this particular region. Since the discovery of FHM-2 [2], a disorder caused by mutations in the ATP1A2 gene at the C1q23 locus, this region has been the focus of intense genetic research for the more common types of migraine. Research on migraine is not only of interest at this locus, other genetic diseases and neurological conditions have also been associated with this region. Family linkage studies conducted by Gardner et al. originally implicated an additional FHM susceptibility locus within a broad region (44 cM) on chromosome 1q31 [16]. Independent research carried out by Ducros et al. indicated a second FHM locus at 1q21-23, approximately 30 cM centromeric to the region reported by Gardner et al [17]. Further investigation of these FHM susceptibility regions has subsequently implicated a specific ATPase gene ATP1A2 on chromosome 1q23. The gene has been identified as having causal mutations in some FHM pedigrees with the locus now defined as FHM2. This region as well as the 1q31 region may also be involved typical migraine with studies suggesting that 1q31 may also be implicated in MO or MA susceptibility in a 82 independent pedigrees [3]. This study investigated markers within both regions 1q23 and 1q31 for involvement in typical migraine. We tested polymorphisms from the KCNJ9, KCNJ10 and FasL genes (markers that had previously been tested in a number of other genetic disorders, including Multiple Sclerosis (MS) [18]).

The CA repeat polymorphism located, upstream of the FasL gene, has previously been found to be associated with MS, in Spanish and American populations [18]. In this study, as well as ours, Zayas et al, 2001 found that the polymorphism consisted of two main alleles, 14 (allele A) and 13 (allele B) repeats, with three others of 11, 12 and 15 repeats having a minor allele frequencies of < 1% in the populations [18]. Therefore the two main alleles were investigated in the multiple sclerosis study as well as in this migraine study. From their results, Zayas et al, 2001 [18] suggested that chromosomes with the B allele have a genetic background that reduces susceptibility to MS. In this report, the CA repeat polymorphism (FasL) showed no significant difference between control and migraine groups (nor for any subtype of migraine, P > 0.05). The FasL gene encodes a protein FasL, which interacts with a transmembrane protein of the tumor necrosis factor receptor superfamily to induce apoptosis in susceptible cells [19]. Neuronal apoptosis engages the p55 protein or MMP-1 (membrane protein palmitoylated 1) in the caspase pathway [20]. Despite a potential neurological role, our results provided no evidence for a role for this FasL genetic variant in migraine.

The KCNJ9 SNP (rs2494211) has been previously associated with type 2 diabetes in Pima Indians [21], and the T allele of the KCNJ10 SNP (rs1130183) showed a significant association with seizure resistance in the common forms of focal and generalized epilepsy patients, suggesting that this variation is related to general seizure susceptibility in humans [22]. As reported in Table 3, the tested SNPs contained within the KCNJ9 and KCNJ10 genes were not significantly associated with migraine or its subtypes (MA, MO) with chi-square results producing *P *values greater than 0.05 for most analyses. However, KCNJ10 genotypic outcomes showed an interesting result (χ^2 ^= 7.65, P = 0.02), that may be worth exploring in a larger, independent population. Our previous study also reported no association between another KCNN3gene (a neuronal small conductance calcium-activated potassium channel localised close to ATP1A2 on C1q23 region) and migraine [4]. KCNJ10 codes for one type of G protein activated inward rectifier potassium (GIRK) type 3 or channel Kir 3.3. These channels have been previously showed stably interacting with 3 different metabotropic receptors (D2 and D4 dopaminergic receptors and the beta 2 adrenergic receptor) by co-precipitation in cell culture. The stable complex formed by G protein-coupled receptors and their effectors exist also in living cells and indicate a physiological relevance [23]. The existence of dopaminergic hypersensitivity in migraine is recognised on pharmacological basis [24], and some studies reported a genetic association between migraine and molecular variations in DA genes as D2 dopaminergic receptor [25] and D4 dopaminergic receptor [26]. It may be interesting to study other actors of this pathway and analysing the genes coding for the regulator of G protein signalling (RGS), more precisely RGS 7, 4, 21 and 21 genes localised in C1q 23.1–31.2 region.

From the three other genes tested on chromosome 1, SNPs were carefully scrutinized and selected to be the most informative genetic markers for these genes, as no previous, published, case-control studies had been performed on these genes. The SNPs contained within the ATP1A4 (rs 6427504) and CASQ1 (rs 3747623) genes and also located on C1q23, did not show any association with migraine or its subtypes with *P *values being greater than 0.05 (Table 3). The ATP1A4 is a subunit of the sodium/potassium ATPase gene, which has been recently associated with a subtype of migraine, FHM [2,27]. However, several reports studying ATP1A2 polymorphisms did not find any involvement in MA [28,29], suggesting a distinct genetic origin for the subtypes of migraine (FHM and MA). The ATP1A2 gene encodes the catalytic subunit of ATPase, co-transporter of ions sodium and potassium, which is abundant in both neurons and astrocytes [30]. This transmembrane protein is localised at the same region than of the Na^+^/Ca^2+ ^exchanger in neurons and astrocytes, demonstrated by immunocytochemistry [31]. This coupled activity of two ion transport systems might be the origin of the pathogenic mechanism leading migraine in FHM. Previous report about FHM (type 1), is caused by mutations contained within the calcium channel gene CACNA1A (localized at C19p13). CACNA1E (C1q31) is subunit of R-type voltage-dependent calcium channel, and the genetic sequence similarities between CACNA1A and CACNA1E are 61% identical in humans. SNP (rs 704326) localised within the CACNA1E gene was investigated in this study but no significant association was found for this polymorphism in total migraine *versus *controls nor in the MA subtype *versus *controls (Table 3). This reinforces the theory that FHM and MA are two different genetic entities.

## Conclusion

Overall our studies investigating potential migraine candidate genes in the chromosome 1q23 and 1q31 regions did not provide evidence implicating any of the tested variants of the ATP1A4, CASQ, KCNJ9 and 10, FasL, CACNA1AE genes in migraine. It is possible that other genetic markers located in these genes and other candidate genes from these genomic regions may be implicated in migraine, but further studies are needed to clarify this possibility. Furthermore, as the power of an association study is affected not only by sample size, but other unknown factors such the strength of the association or degree of difference between the case and control subjects, as well as the genetic effect (ie. penetrance) [32], the possibility of false negative results should not be ruled out.

## Competing interests

The author(s) declare that they have no competing interests.

## Authors' contributions

FF and RC were responsible for undertaking all the experiments and the analysis of data. LG conceived and coordinated the study, revised the manuscript. All authors read and approved the final manuscript.

## Pre-publication history

The pre-publication history for this paper can be accessed here:


